# Gastric adenocarcinoma burden, trends and survival in Cali, Colombia: A retrospective cohort study

**DOI:** 10.3389/fonc.2023.1069369

**Published:** 2023-03-07

**Authors:** Luis Gabriel Parra-Lara, Juan Camilo Falla-Martínez, Daniel Francisco Isaza-Pierotti, Diana Marcela Mendoza-Urbano, Andrés R. Tangua-Arias, Juan Carlos Bravo, Luis Eduardo Bravo, Ángela R. Zambrano

**Affiliations:** ^1^ Facultad de Ciencias de la Salud, Universidad Icesi, Cali, Colombia; ^2^ Centro de Investigaciones Clínicas, Fundación Valle del Lili, Cali, Colombia; ^3^ Departamento de Patología, Fundación Valle del Lili, Cali, Colombia; ^4^ Registro Poblacional de Cáncer de Cali, Departamento de Patología, Facultad de Salud, Universidad del Valle, Cali, Colombia; ^5^ Servicio de Hemato-Oncología, Departamento de Medicina Interna, Fundación Valle del Lili, Cali, Colombia

**Keywords:** gastric cancer, lymphadenectomy, gastrectomy, gastric cancer survival, adjuvant chemotherapy, Helicobacter pylori

## Abstract

**Background:**

Gastric adenocarcinoma (GA) has changed in recent decades. Cancer estimates are often calculated from population-based cancer registries, which lack valuable information to guide decision-making (clinical outcomes). We describe the trends in clinical practice for GA using a hospital-based cancer registry over a timespan of 15 years.

**Methods:**

A retrospective cohort study was conducted. Data were gathered from adults diagnosed and treated for GA at Fundación Valle del Lili (FVL), between 2000 and 2014, from the hospital’s own cancer registry and crossed with Cali’s Cancer Registry. Additional data were obtained directly from clinical records, pathology reports and the clinical laboratory. Patients younger than 18 years and those for whom limited information was available in the medical history were excluded. A survival analysis was conducted using Kaplan-Meier method.

**Results:**

A total of 500 patients met eligibility criteria. Median age was 64 years (IQR: 54-74 years), 39.8% were female, 22.2% were at an early stage, 32.2% had a locally advanced disease, and 29% a metastatic disease, 69% had intestinal subtype, 48.6% had a positive *H. pylori* test, 85.2% had a distal lesion, 62% underwent gastrectomy, 60.6% lymphadenectomy, and 40.6% received chemotherapy. Survival at 5 years for all cases was 39.9% (CI 95% 35.3-44.5). Survival decreased over time in all groups and was lower in age-groups <39 and 60-79 with either locally advanced or metastatic disease. Prognostic factors that were significant in the Cox proportional-hazards model were late stages of the tumor (locally advanced: HR=2.52; metastatic: HR=4.17), diffuse subtype (HR=1.40), gastrectomy (subtotal: HR=0.42; total: 0.44) and palliative chemotherapy (HR=0.61).

**Conclusions:**

The treatment of GA has changed in recent decades. GA survival was associated with clinical staging, diffuse subtype, gastrectomy and palliative chemotherapy. These findings must be interpreted in the context of a hospital-based study.

## 1 Introduction

Worldwide, stomach cancer (SC) remains among the most common neoplasms and is one of the leading causes of cancer deaths (1). The pathophysiology of SC is multifactorial and complex. Although most people with *H. pylori* infection will not develop SC, *cagA* positive genotypes (which cause chronic inflammation and hypochlorhydria) served for better understanding pathophysiologic processes leading to the development of SC (2).

Therapeutic strategies aim to maintain quality of life while improving survival rates. Surgery is the mainstay of treatment for resectable disease, especially the spleen and pancreas-sparing D2 lymphadenectomy, whereas unresectable disease is preoperatively treated with a wide variety of nonsurgical options (3, 4). Several advances in recent years have influenced the approach to SC from the therapeutic and diagnostic standpoints. As an example, serum CD26 (an ectoenzyme with dipeptidyl peptidase 4 activity) levels have been proposed as a potential screening tool (5). Furthermore, molecular targets are being actively investigated with different pharmacologic strategies (i.e., anti-VEGF/VEGFR agents, anti-EGFR therapies, HER2 targeting agents, PI3K-AKT-mTOR targeted therapy, HGF-c-Met pathway inhibitors, and FGFR inhibitors) aiming to improve the prognosis, especially in unresectable disease (6).

Although The American Joint Committee on Cancer (AJCC)/Union for International Cancer Control (UICC) tumor, node, metastasis (TNM) staging system classification has changed across its different editions, it defines SC as resectable or unresectable, an important distinction for therapeutics, and is widely accepted as an accurate prognostic factor (3). Global geographic differences in SC epidemiology have influenced the adoption of different classification systems. For instance, in Japan, as compared to Western countries, higher survival rates are thought to be due to the implementation of intense screening programs (4). Such observations lead to consider differences in the pathophysiologic processes governing the development of SC. In consequence, molecular classifications like those proposed by The Cancer Genome Atlas (TCGA) and Asian Cancer Research Group (ACRG) approach SC from the pathophysiologic perspective, aiming to guide decision-making in a more patient-centered fashion as part of a precision medicine strategy (3).

SC can be classified by the anatomical location of the primary tumor as cardia and non-cardia (7). SC is most importantly caused by gastric adenocarcinoma (GA), accounting for roughly 90% of the cases (8). Lauren’s histopathological classification is widely reported in the literature and recognizes GA as diffuse and intestinal subtypes, each with its own therapeutic and prognostic value (8–11). However, the World Health Organization (WHO) classification recognizes a more varied number of histopathological subtypes (12).

Cancer registries provide the data-driven foundation for cancer control efforts. There are few data on the survival of GA in Colombia. Data on clinical stage and therapeutic interventions are not included in population-based cancer registries (PBCRs). These covariates are very helpful in cancer because they allow for data-driven decision-making policies and population-based interventions oriented to improve survival rates. Some hospital-based cancer registries (HBCRs) include such covariates and have been implemented in Cali, Colombia (13). The objective of this study was to report our experience with the Fundación Valle del Lili’s HBCR. The focus is to report demographic and clinical characteristics of patients with SC, specifically with GA, treated in the period of 2000-2014 at Fundación Valle del Lili, and to estimate the overall survival at 5-year intervals.

## 2 Materials and methods

### 2.1 Study design

This was a retrospective cohort study from GA patients seen at Fundación Valle del Lili, a quaternary-level of care private non-profit academic medical center located in Cali, Colombia. Retrospective data were obtained from the hospital-based cancer registry (HBCR). This HBCR includes demographic, tumor classification, treatment, and follow-up data.

Patients with histopathological diagnoses of GA between 2000 and 2014 from the HBCR were included. Patients <18 years of age, incomplete information, staged as *in situ*, multiple primary tumors, and those diagnosed and/or treated in another institution were excluded. Identified cases were matched with the RPCC database, which has been validated elsewhere (14).

According to the institutional protocol, gastroenterologists perform a sampling of 5 to 30 biopsies per case. Broad sampling is applied in cases of early injuries. In gastrectomy cases, twenty paraffin blocks are analyzed on average.

Systemic therapy (chemotherapy) and surgical management were carried out according to the recommendations of the current National Comprehensive Cancer Network (NCCN) guidelines for each period. The indication for total or subtotal gastrectomy was defined according to the anatomical location of the tumor, surgical margins, tumor biology, and the patient age.

### 2.2 Variables, potential biases, and missing data

Sociodemographic (age, sex, type of health insurance, *H. pylori* screening), disease classification (Lauren’s histopathological classification, disease staging, anatomical location of the tumor), treatment (type of surgical management, type of lymphadenectomy, type of medical therapy), and follow-up variables (follow-up time, mortality) are described.

To visualize the changing trends in surgical management, the number of patients diagnosed with GA over time were plotted along with the respective proportions of those who underwent surgical treatment, types of gastrectomy and lymphadenectomy.

The time from histopathological diagnosis to death was calculated from the available dates in the datasets. As per matching the HBCR and the RPCC, the IARC/WHO International Classification for Diseases in Oncology 3rd Edition (ICD-O-3), last patient contact, and mortality data were obtained. This also served as a quality check to enhance data integrity by allowing to observe a more accurate follow-up time when the last patient contact occurred in another institution and to decrease the amount of censored data from the HBCR. However, it did not eliminate the potential for unobserved follow-up time and mortality by the timeframe in which the data were retrieved from the cancer registries. Missing data were described for disease staging.

### 2.3 Statistical analysis

A descriptive analysis of the sociodemographic and clinical variables was performed using measures of central tendency (mean or median) and dispersion (standard deviation or interquartile range). The data distribution was evaluated with the Shapiro-Wilk test.

Survival analysis was performed using the Kaplan-Meier method. Survival was calculated using the date of diagnosis and the date of death or the last day of follow-up (the last day of hospital care or the date of last contact recorded by the RPCC; the most recent date was used). Kaplan-Meier curves were plotted for subgroup analysis at 12, 36 and 60 months, and comparisons were made by means of the log-rank test. Also, we compared overall survival according to cancer staging and age groups from the national registry of the *Japanese Gastric Cancer Association (JGCA)* (15). Histopathological classification, disease stage, type of lymphadenectomy, and anatomical location were analyzed.

Variables with p<0.2 in the univariate analysis were subjected to Cox proportional-hazards regression analysis with p< 0.05 to evaluate the effects of the prognostic factors. The proportionality assumption was verified using the model-specific test and Cox-Snell residuals (**Supplementary File**). Data analyses were generated with STATA^®^ (Version 14.0, StataCorp LP, College Station, TX).

## 3 Results

A total of 732 patients were identified between the years 2000 and 2014. A total of 232 patients were excluded due to incomplete information (n = 38), other anatomical localization (n = 80), diagnosis in other period (n = 74), staged as *in situ* (n = 20), and multiple primary tumors (n = 20). A total of 500 patients were included in the study after fulfilling the selection criteria.

Demographic and clinical data were summarized in **Table 1**. The frequency of cases by JGCA age groups was 7.4% (≤39 y), 28% (40-59 y), 52.6% (60-79 y) and 12% (≥80 y). The majority were from male sex (60%). Seventy-three-point eight percent received treatment (chemotherapy, surgery or radiotherapy). Sixty-two percent was treated with a surgical procedure, 61% with lymphadenectomy and 41% with any chemotherapy regimen. The median follow-up was 14.45 months (IQR=2.85-44.75 months).

**Table 1:** Characteristics of the included patients (n=500).

Characteristics	Total cases, n=500	Alive, n=202	Deaths, n=298
Age at diagnosis, n (%)			
≥50 years	410 (82)	160 (39)	250 (61)
Gender, n (%)			
Male	301 (60)	125 (41.5)	176 (58.5)
Clinical stage, n (%)			
Early	111 (22)	75 (67.6)	36 (32.4)
Locally advanced	161 (32)	71 (44.1)	90 (55.9)
Metastatic	145 (29)	24 (16.5)	121 (83.5)
No data	83 (17)	32 (38.5)	51 (61.5)
Lauren classification, n (%)			
Intestinal	345 (69)	155 (44.9)	190 (55.1)
Diffuse	155 (31)	47 (30.3)	108 (69.7)
Location, n (%)			
Proximal	74 (15)	27 (36.5)	47 (63.5)
Distal	426 (85)	175 (41.1)	251 (58.9)
Positive *H. pylori* test, n (%)			
Yes	43 (9)	22 (51.2)	21 (48.8)
No	457 (91)	180 (39.4)	277 (60.6)
Gastrectomy, n (%)			
No	190 (38)	45 (23.7)	145 (76.3)
Subtotal	154 (31)	83 (53.9)	71 (46.1)
Total	156 (31)	74 (47.4)	82 (52.6)
Lymphadenectomy			
No	176 (35)	41 (23.3)	135 (76.7)
D1	70 (14)	46 (65.7)	24 (34.3)
D2	233 (47)	104 (44.6)	129 (55.4)
No data	21 (4)	11 (52.4)	10 (47.6)
Chemotherapy, n (%)			
No	297 (59)	121 (40.7)	176 (59.3)
Adjuvant	140 (28)	63 (45)	77 (55)
Neoadjuvant	18 (4)	7 (38.9)	11 (61.1)
Palliative	45 (9)	11 (24.4)	34 (75.6)

**Figure 1:**
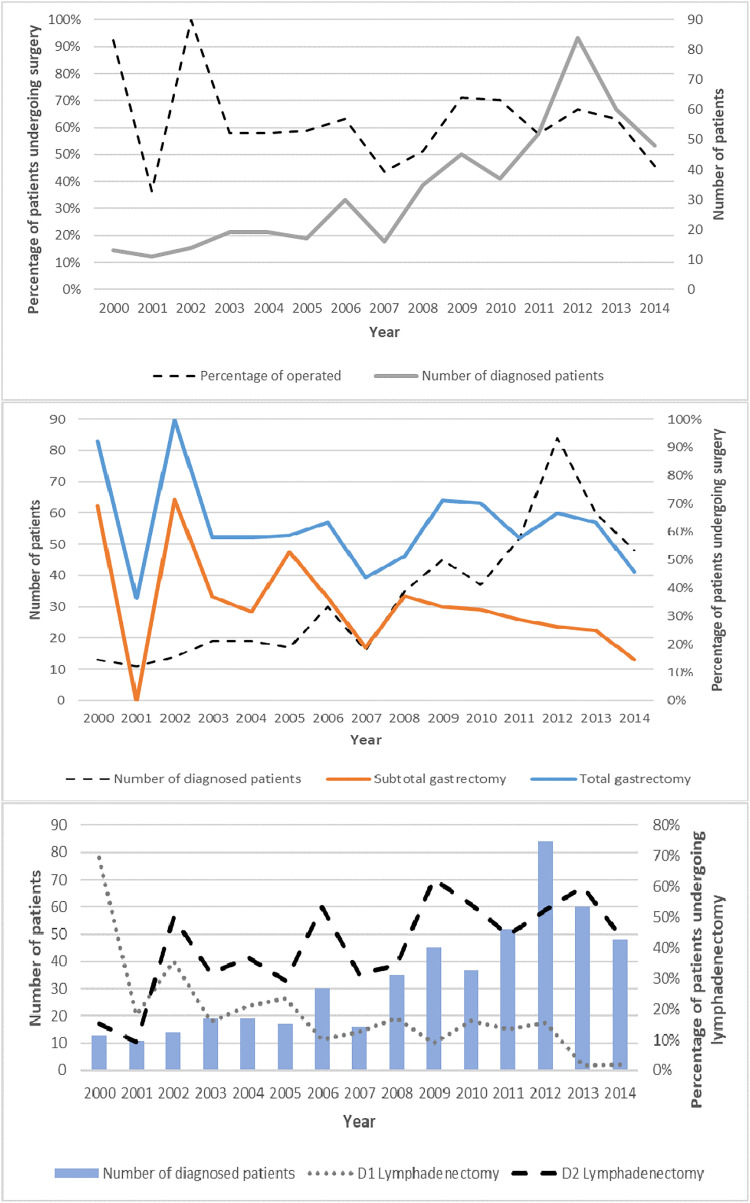
Trend curves. Number of diagnosed cases, percentage of operated patients, type of gastrectomy and type of lymphadenectomy, period 2000-2014.

**Figure 1** presents the trends of GA diagnoses, proportion of patients who underwent surgical management, types of surgery and lymphadenectomy. Over time, the number of diagnoses increased, whereas the proportion of patients who underwent surgical management was maintained between 40% and 70% after 2003. The most frequent type of surgery was the total gastrectomy, which increased in proportion after 2008 as compared to subtotal gastrectomy. A similar trend was observed for types of lymphadenectomy, where less conservative management increased in proportion over time. In 2000 the most frequent type of lymphadenectomy was D1. Later, in 2002, D2 lymphadenectomy began increasing in popularity until 2013 when it became virtually the only type practiced.

**Figure 2:**
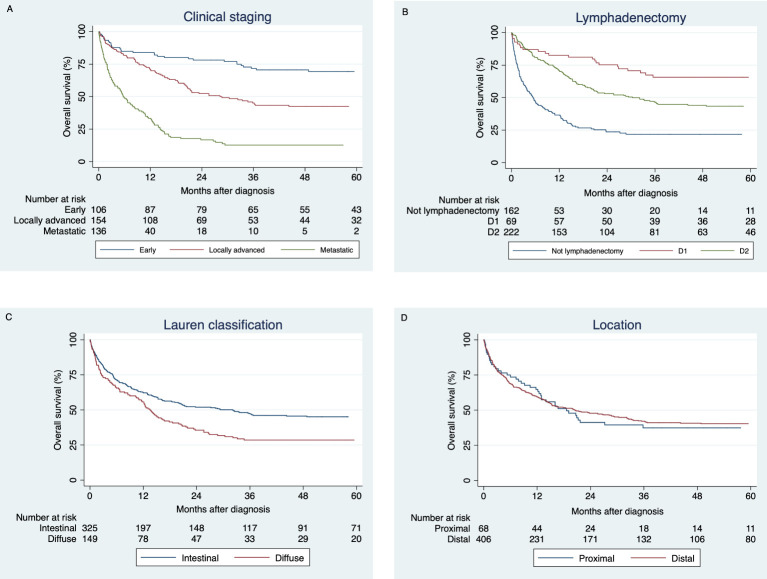
5-year survival function estimations by the Kaplan-Meier method comparing: **(A)** Early, locally advanced and metastatic stages. **(B)** Lymphadenectomy (D1, D2 or without intervention). **(C)** Lauren classification (intestinal and diffuse subtypes). **(D)** Location (proximal and distal).

Survival estimates at 12, 36 and 60 months from diagnosis are shown in **Table 2**. Survival at 5 years for all cases was 39.9% (CI 95% 35.3-44.5). There were statistical differences in the survival for clinical stage (p<0.001), Lauren classification (p=0.001), *H. pylori* infection (p=0.035), gastrectomy (p<0.001), lymphadenectomy (p<0.001) and chemotherapy (p=0.001). No significant differences were found for anatomical location (p=0.777). **Figure 2** presented the Kaplan-Meier curves at 5 years by subgroups.

**Table 2:** Survival estimates at 12, 36 and 60 months from diagnosis using Kaplan-Meier method.

Variables	Survival rate (CI 95%)			p-value^†^
	12 m	36 m	60 m	
**All cases**	60.2 (55.6-64.5)	41.1 (36.5-45.7)	39.9 (35.3-44.5)	
Age group				
<50 y	62.9 (51.6-72.3)	46.3 (34.9-56.9)	46.3 (34.9-56.9)	0.347
≥50 y	59.3 (54.3-64.1)	40.0 (34.9-45.0)	38.5 (33.4-43.5)	
Clinical stage				
Early	83.0 (74.4-88.9)	71.7 (61.8-79.4)	69.3 (59.2-77.4)	<0.001
Locally advanced	70.6 (62.6-77.1)	44.2 (35.8-52.3)	42.4 (34.1-50.5)	
Metastatic	32.4 (24.5-40.5)	12.7 (7.3-19.5)	12.7 (7.3-19.5)	
No data	52.1 (40.4-62.5)	39.4 (28.3-50.3)	39.4 (28.3-50.3)	
Lauren classification				
Intestinal	62.2 (56.7-67.3)	46.9 (41.2-52.4)	45.1 (39.4-50.7)	0.001
Diffuse	55.0 (46.6-62.7)	28.5 (21.3-36.2)	28.5 (21.3-36.2)	
Location				
Proximal	63.2 (50.6-73.5)	37.4 (25.6-49.2)	37.4 (25.6-49.2)	0.777
Distal	59.4 (54.4-64.0)	41.7 (36.7-46.7)	40.3 (35.3-45.3)	
Positive *H. pylori* test				
Yes	72.9 (55.5-84.4)	58.1 (40.2-72.3)	55.0 (37.2-69.7)	0.035
No	58.8 (54.0-63.3)	39.3 (34.6-44.1)	38.6 (33.8-43.4)	
Gastrectomy				
No	35.9 (28.7-43.1)	22.2 (16.0-29.0)	22.2 (16.0-29.0)	<0.001
Subtotal	76.2 (68.4-82.3)	54.8 (46.1-62.6)	54.8 (46.1-62.6)	
Total	71.1 (63.1-77.7)	48.3 (39.8-56.3)	45.2 (36.6-53.5)	
Lymphadenectomy				
No	35.9 (28.5-43.4)	21.8 (15.4-28.9)	21.8 (15.4-28.9)	<0.001
D1	81.2 (69.8-88.6)	65.7 (52.9-75.8)	65.7 (52.9-75.8)	
D2	70.4 (63.9-76.0)	46.0 (39.0-52.7)	43.4 (36.4-50.2)	
No data	57.1 (33.8-74.9)	52.0 (29.1-70.6)	52.0 (29.1-70.6)	
Chemotherapy				
No	52.8 (46.8-58.5)	42.7 (36.7-48.5)	41.7 (35.7-47.5)	0.001
Adjuvance	76.6 (68.5-83.0)	45.2 (36.2-53.8)	43.0 (34.1-51.7)	
Neoadjuvance	75.0 (46.3-89.8)	33.6 (9.9-59.8)	33.6 (9.9-59.8)	
Palliative	46.5 (31.0-60.5)	15.9 (5.5-31.3)	15.94 (5.5-31.3)	

^†^Log-rank test.

**Table 3** shows the prognostic factors in the Cox proportional-hazards model, finding that late tumor stages, such as locally advanced and metastatic, increase the risk of dying from this condition approximately two and four times, respectively. Diffuse histological subtype increases the risk of dying more than one time. Important protective factors were subtotal and total gastrectomy and chemotherapy with palliative intent. A factor studied that did not contribute with significant information was positive *H. pylori* test.

**Table 3:** Prognostic factors for gastric adenocarcinoma.

Variable	HR	IC 95%
Clinical stage		
Early	1^†^	
Locally advanced	2.52	1.62-3.90
Metastatic	4.17	2.58-6.73
No data	1.59	0.93-2.72
Lauren classification		
Intestinal	1^†^	
Diffuse	1.40	1.09-1.80
Positive *H. pylori* test		
Yes	0.75	0.45-1.26
No	1^†^	
Gastrectomy		
No	1^†^	
Subtotal	0.42	0.27-0.65
Total	0.44	0.28-0.68
Chemotherapy		
No	1^†^	
Adjuvance	0.82	0.58-1.17
Neoadjuvance	0.60	0.30-1.19
Palliative	0.61	0.40-0.92

^†^Reference category.

Survival at 5 years for disease stage by age groups from the JGCA was summarized in **Table 4**. The best probability of survival was for patients with an early stage between 40-59 years with a 5-year overall survival of 69.0% (IC 95% 47.5-83.2), while the worst probability of survival occurred in patients with a metastatic stage, aged ≤39 years with a 5-year overall survival of 10.3% (IC 95% 0.7-35.5).

**Table 4:** 5-year survival according to cancer staging and age groups from the *Japanese Gastric Cancer Association*.

	Early n=111	5-y OS (CI 95%)	Locally advanced n=161	5-y OS (CI 95%)	Metastatic n=145	5-y OS (CI 95%)	No data n=83	5-y OS (CI 95%)
Age group								
≤39	5	–	13	50.0 (20.9-73.6)	14	10.3 (0.7-35.5)	5	40.0 (5.2-75.3)
40-59	26	69.0 (47.5-83.2)	47	53.7 (36.5-68.2)	48	14.1 (5.2-27.4)	19	44.6 (20.4-66.3)
60-79	64	67.1 (53.3-77.7)	91	35.7 (25.3-46.2)	67	11.1 (4.4-21.3)	41	38.4 (22.9-53.6)
≥80	16	61.1 (24.5-84.2)	10	44.4 (13.6-71.9)	16	14.3 (2.4-36.3)	18	38.1 (16.6-59.5)

5-y OS: 5-years overall survival.

## 4 Discussion

This is the first study conducted on GA from the hospital-based cancer registry data in Latin America. This study showed that GA overall survival at 5 years was 39.9% in our hospital setting. Differences in survival were observed according to clinical stage, Lauren’s classification, H. pylori infection, surgical management, and systemic therapy.

SC is a disease of public health concern globally (1). The burden is higher in males than in females, and in Eastern and South-Central Asia, Eastern Europe, and South America as compared to other parts of the world (1). Risk factors include sociodemographic and genetic characteristics, diet and excess body weight, alcohol and tobacco consumption, antibiotics use, *H. pylori* infection, dysbiosis of the gastric microbiome, autoimmune gastritis, gastric ulcers, and gastroesophageal reflux disease, among others (11). Several screening techniques exist, although the value of mass screening is controversial and depends on the incidence of the disease (16, 17). For instance, Japan has adopted a mass screening strategy showing good results (18), whereas in countries with lower incidence, such as the United States (US), this may not be cost-effective (16).

In FVL, between 2000 and 2014, most GA cases in patients older than 18 years were distributed within the 60-79 years. This is consistent with data from United States, Europe, Latin America, Asia-Pacific, and the globe (19, 20). Nevertheless, in the Indian subcontinent and North Africa most cases are distributed within the 50-59 years of age group (19). These similarities and differences may reflect the interplay between sociodemographic characteristics and risk factors within each of these populations.

Intestinal subtype predominated in our patients; this may be related to the characteristics of the analyzed population. It has been reported that intestinal subtype tumors occur more frequently in elderly patients, males, and predominantly in distal locations (21). Survival curves between intestinal and diffuse subtype tumors were statistically different; probably because the intestinal subtype presents less lymphovascular invasion and a lower recurrence rate (21).

Furthermore, the most important clinical characteristic used for decision-making in practice is the GA disease stage, defined by the extension of the tumor as localized (confined to the mucosa and submucosa), locally invasive (extending to the muscularis propria and beyond), and metastatic (compromising lymph nodes and distant tissues) (22–24). Localized GA is considered early disease, whereas locally invasive and metastatic are considered late disease (25). These distinctions are also aligned with the American Joint Cancer Committee/Union for International Cancer Control (AJCC/UICC) guidelines disease staging system (26). The proportion of early-stage tumors was slightly higher than observed in Latin America. In this region, during the period 2004-2008, the percentage of early-stage tumors was 16.1% (19).

Before the year 2000, gastric cancer was considered refractory to chemotherapy, and surgery was accepted as the only treatment with curative intent (27). This changed in 2001 when a clinical trial showed that receiving surgery plus adjuvant chemoradiation was associated with increased survival, albeit with significant toxicity (28). Then, the MAGIC study, in 2006, showed the benefit in progression-free and overall survival with perioperative chemotherapy regime (before and after surgery) with epirubicin, cisplatin, and fluorouracil (ECF) (29). In 2012, the CLASSIC study reported improved 3-year disease-free survival in patients who received adjuvant capecitabine plus oxaliplatin (XELOX) after curative D2 gastrectomy, compared with a surgery-alone group (30). Subsequently, the FLOT4 study showed the superiority of docetaxel-based triplet FLOT (fluorouracil plus leucovorin, oxaliplatin and docetaxel) in perioperative chemotherapy in patients with locally advanced, resectable tumors, compared to the scheme with ECF proposed in the MAGIC study (31).

The rapid evolution of the last two decades of GA treatment has caused health institutions to frequently change their care protocols to keep pace with new evidence as it is published. In this study, the way in which patients were included in the chemotherapy subgroup (higher proportion of patients who did not receive chemotherapy regime, or who received it as adjuvant therapy) reflects the transition in the period from 2000 to 2014 in institutional protocols from isolated surgical management to the incorporation of adjuvant therapy and finally perioperative chemotherapy. Additionally, considering that in our hospital most of the patients had an advanced clinical stage, the transition to perioperative management with triplet-based chemotherapy has developed over time, which is consistent with the global trend and is justified in the results of clinical trials in recent years.

In this study, the 5-year overall survival was 39.9%. From 2000-2014, 5-year age-standardized net survival (ASNS) was in the range of 10–30% in most countries for gastric cancer, with survivals as high as 60.3% in Japan and 68.9% in Korea, both from the Asian continent (32). In Colombia, for the same period, the ASNS was between 15.4 and 20.9% (32). Our findings show that overall survival in GA is higher than national statistics, which can be explained by the changes implemented in the treatment of patients. There is still a gap compared to Asian countries, which could be related to the implementation of population screening strategies for the early diagnosis of GA.

Complete surgical resection of the primary tumor and regional lymph nodes is the mainstay of GA treatment, except for metastatic disease (33). Although palliative gastrectomy is recognized, chemotherapy is considered first-line treatment for non-curable GA patients (34). For localized and locally invasive disease, indications for total and partial gastrectomy depend on the anatomical location of the primary tumor, histopathological subtype, and disease stage (34).

Regional lymphadenectomy has a positive impact on survival and is routinely performed in addition to the gastrectomy (28, 35, 36). It is categorized depending on the extension of node dissection, being D1 for the perigastric nodes, D2 for the perigastric along with the celiac artery system nodes, and D3 includes Additionally nodes within the porta hepatis and those adjacent to the aorta. The AJCC/UICC guidelines requires the evaluation of at least 16 nodes for accurate disease staging (26).

There was a higher 5-year survival found in patients with D1 lymphadenectomy. Analyzing lymphadenectomy trends (**Figure 1**), we found that during the years 2000-2014, D1 lymphadenectomy was progressively abandoned, and the period ended with the performance of D2 lymphadenectomy in practically all procedures. The possibility arises that during the study period, the patients who were considered more compromised were the same ones who were selected for D2. Other factors that could contribute is the higher short-term mortality in D2, and the benefit of D2 in the Western population has been more consistent in studies that evaluated survival times longer than 5 years (37).

Outcomes related to lymphadenectomy type also depend on the degree of expertise, due to the higher risk of postoperative complications associated with a more extensive dissection. Management in hospitals with high volumes of annual surgery and the performance of the procedure by expert surgeons reduce the risk of early complications (38). Survival results obtained for this transition (during 2000-2014) may be influenced by the previously mentioned confounding factors that need to be explored in our context in future research.

The study had several limitations, many of which are a consequence of its design. First, as a retrospective cohort study, it included data obtained from secondary data sources (medical records and health system databases); consequently, information bias could be present. Second, the study was conducted at a single health center in the city, and therefore, it is not representative of the whole region/country, but it is key in generating new knowledge about GA in our context. Third, attempts to evaluate the impact of the presence or absence of cancer treatment on survival could be confounded by changes that occurred during follow-up, such as changes in the type of surgery, chemotherapy, lymphadenectomy and changes in the patients’ comorbidities.

The strength of this study was the good quality of information regarding the description of cancer, its diagnosis and follow-up (vital status) from the RPCC, which is considered the most important source of descriptive epidemiological information for cancer in Latin America. The inclusion of this information makes this a unique study in the region.

In conclusion, the management of gastric adenocarcinoma has changed in recent decades, impacting survival and clinical outcomes. Survival in our hospital was higher than that reported in the city and the country. There is a transition in the therapeutic approach that is expected to have an impact in the coming years (mainly due to D2 lymphadenectomy and chemotherapy regimens). Screening options should be explored in our population. These findings must be interpreted in the context of a hospital-based study.

## Data availability statement

The raw data supporting the conclusions of this article will be made available by the authors, without undue reservation.

## Ethics statement

The studies involving human participants were reviewed and approved by Comité de Ética en Investigación Biomédica. Written informed consent for participation was not required for this study in accordance with the national legislation and the institutional requirements.

## Author contributions

Conception and design: AZ, JF-M, JB, LP-L. Administrative support: LP-L, DM-U, AT-A. Provision of study materials or patients: JF-M, LP-L, AZ, JB. Collection and assembly of data: LP-L, JF-M. Data analysis and interpretation: All authors. Manuscript writing: All authors. Final approval of manuscript: All authors.
