# Supplementary material for: Gastric adenocarcinoma burden, trends and survival in Cali, Colombia: A retrospective cohort study
Source: Front Oncol. 2023 Mar 7;13:1069369. doi: 10.3389/fonc.2023.1069369 (PMC10028196; doi:10.3389/fonc.2023.1069369)

Supplementary Material

# Supplementary Data

N.A.

# Supplementary Figures and Tables

**Table S1.** Model-specific test.


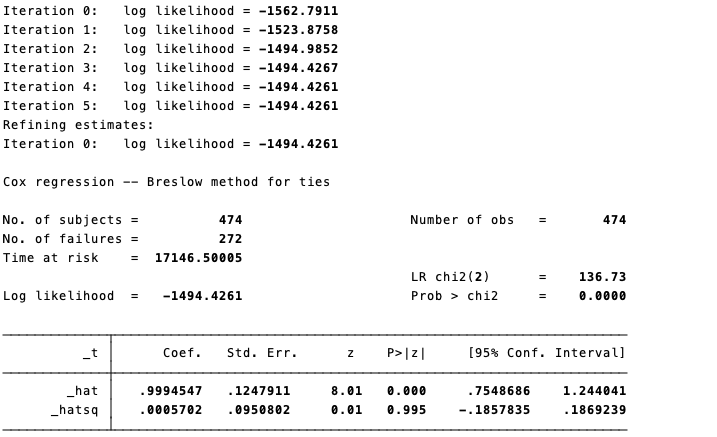


**Figure S1.** Cox-Snell residuals plot.


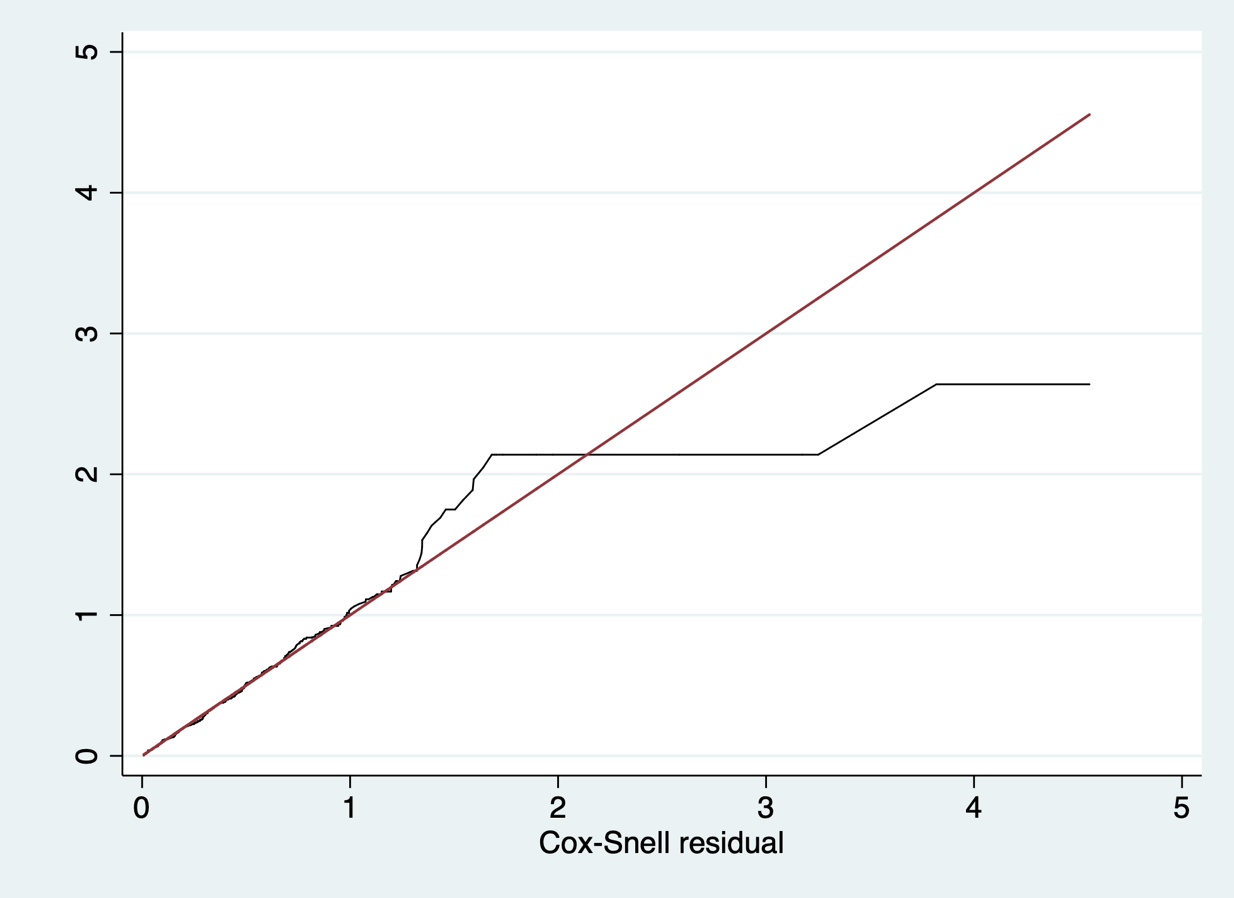

Supplement: Supplementary file 1 [file DataSheet_1.docx]
